# Fibrosis modeling choice affects morphology of ventricular arrhythmia in non-ischemic cardiomyopathy

**DOI:** 10.3389/fphys.2024.1370795

**Published:** 2024-03-18

**Authors:** Lena Myklebust, Mary M. Maleckar, Hermenegild Arevalo

**Affiliations:** Computational Physiology, Simula Research Laboratory, Oslo, Norway

**Keywords:** patient-specific modeling, computational electrophysiology, re-entrant arrhythmia, cardiac fibrosis, non-ischemic cardiomyopathy, late gadolinium enhanced magnetic resonance imaging, electrical heterogeneity, ventricular models

## Abstract

**Introduction:** Patients with non-ischemic cardiomyopathy (NICM) are at risk for ventricular arrhythmias, but diagnosis and treatment planning remain a serious clinical challenge. Although computational modeling has provided valuable insight into arrhythmic mechanisms, the optimal method for simulating reentry in NICM patients with structural disease is unknown.

**Methods:** Here, we compare the effects of fibrotic representation on both reentry initiation and reentry morphology in patient-specific cardiac models. We investigate models with heterogeneous networks of non-conducting structures (cleft models) and models where fibrosis is represented as a dense core with a surrounding border zone (non-cleft models). Using segmented cardiac magnetic resonance with late gadolinium enhancement (LGE) of five NICM patients, we created 185 3D ventricular electrophysiological models with different fibrotic representations (clefts, reduced conductivity and ionic remodeling).

**Results:** Reentry was induced by electrical pacing in 647 out of 3,145 simulations. Both cleft and non-cleft models can give rise to double-loop reentries meandering through fibrotic regions (Type 1-reentry). When accounting for fibrotic volume, the initiation sites of these reentries are associated with high local fibrotic density (mean LGE in cleft models: p < 0.001, core volume in non-cleft models: *p* = 0.018, negative binomial regression). In non-cleft models, Type 1-reentries required slow conduction in core tissue (non-clefts_*c*_ models) as opposed to total conduction block. Incorporating ionic remodeling in fibrotic regions can give rise to single- or double-loop rotors close to healthy-fibrotic interfaces (Type 2-reentry). Increasing the cleft density or core-to-border zone ratio in cleft and non-cleft_*c*_ models, respectively, leads to increased inducibility and a change in reentry morphology from Type 2 to Type 1.

**Conclusions:** By demonstrating how fibrotic representation affects reentry morphology and location, our findings can aid model selection for simulating arrhythmogenesis in NICM.

## 1 Introduction

Sudden cardiac death (SCD) is a leading cause of mortality worldwide (Wong et al., 2019), often resulting from ventricular arrhythmia. Patients with non-ischemic cardiomyopathy (NICM) - cardiac dysfunction not caused by impaired coronary flow - are at increased risk of these dangerous arrhythmias. Even with intervention, patients with NICM have a 3.5 years cardiac mortality rate of 4%, where a fourth of cases is due to SCD (Narins et al., 2022). However, the arrhythmic mechanisms in these patients are not well understood, and thus risk stratification and treatment planning remain challenging.

Characterised by an accumulation of collagen, fibrosis disrupts the electrical coupling between myocytes, effectively slowing or blocking conduction (Nguyen et al., 2014). In turn, these factors are known to promote the formation of reentrant arrhythmia (Rensma et al., 1988). Cardiac fibrosis can be detected as areas of late gadolinium enhancement (LGE) on cardiac magnetic resonance (CMR) images, making it a feasible metric for risk stratification. In the recent years, LGE-detected fibrosis (from here on referred to as LGE) has emerged a strong risk predictor for patients with NICM, supporting its use in treatment planning (Leyva et al., 2017; Gutman et al., 2019; Theerasuwipakorn et al., 2023). The arrhythmic risk associated with LGE does, however, not only depend on it being present, but also on its extent, location, pattern and texture (Nguyen et al., 2014; Halliday et al., 2019; Zorzi et al., 2016). Given the complexity, the optimal strategy for using LGE to diagnose patients with NICM remains undetermined.

In clinically-directed computational models of the heart and its tissues, LGE can be represented as regional changes in cellular membrane currents and tissue conductivities. We refer to models with only such changes as non-cleft models. In non-cleft models, fibrotic tissue may be classified as either border zone or core scar, where the border zone is assigned pathophysiological ionic remodeling and a reduction in conductivity, while the core is non-conductive. This method, a standard approach for modeling ischemic cardiomyopathy (Arevalo et al., 2016), has also been used to predict arrhythmic events in NICM (Cartoski et al., 2019; Shade et al., 2020; O’Hara et al., 2022). However, due to differences in fibrotic morphology, methods well suited for modeling ischemic cardiomyopathy may not be equally appropriate for NICM. In ischemic cardiomyopathy, fibrosis often involves a dense core with a surrounding region of intermediate remodeling (Schelbert et al., 2010). In contrast, NICM patients present with a large variety of patterns: from commonly patchy, diffuse and interstitial fibrosis to, more rarely, compact fibrosis (Glashan et al., 2018).

As an alternative to the non-cleft method, the heterogeneity of fibrosis may be represented by creating small no-flux boundaries, or clefts, in the models determined by the LGE intensity (cleft models). Clefts may be used to create various fibrotic architectures, such as interstitial or patchy, depending on the cleft density and pattern. This approach has already been used to model arrhythmia in NICM patients in 2D and in slabs of 3D ventricular tissue (Costa et al., 2013; Balaban et al. 2018; Balaban et al. 2020). A following clinical validation of the method in 2D shows that simulated reentry is associated with risk of arrhythmia in NICM patients (Balaban et al., 2021).

While both non-cleft and cleft models have been used to model reentry in fibrosis, few studies have focused on comparing the two approaches. 2D simulations suggest that only discrete structures can fully capture the effects of fibrosis on reentry (Gokhale et al., 2017). Others have investigated the influence of modeling strategies in patient-specific bilayer atrial models (Roney et al., 2016). However, how well these methods may describe arrhythmic mechanisms in the context of 3D fibrosis, including in NICM patients, is thus far unclear. The goal of this study is to assess and compare these modeling approaches in the case of NICM using patient-specific 3D ventricular models.

Building on the above-mentioned recent work, we created ventricular models with different fibrotic representations. Of specific interest is to compare models with fibrotic clefts to simpler, non-cleft models, as described above. We show that both modeling method and parameter choice affect reentry in terms of reentry inducibility, location and morphology. Understanding the impact of modeling choice on simulated arrhythmias leads the way for determining an optimal approach for computationally-supported risk stratification in NICM patients.

## 2 Materials and methods

### 2.1 Patient cohort

The DANISH (Defibrillator Implantation in Patients With Nonischemic Systolic Heart Failure) trial evaluated prophylactic ICD implantation in patients with symptomatic, non-ischemic systolic heart failure (NYHA class II-IV) across Denmark (Køber et al., 2016). Here, we leveraged anonymized LGE-CMR images of six patients, obtained through the DANISH study and provided by Rigshospitalet in Copenhagen, DK. All images were of the short-axis plane, using 8 mm slice thickness and 1.3–1.5 mm resolution.

### 2.2 Segmentation

We manually delineated the left and right ventricular epi- and endocardial contours. To identify fibrotic regions, we used a fully-automated algorithm which uses Expectation Maximization to classify image intensity. The method is implemented in Segment (Heiberg et al., 2010) and described in (Engblom et al., 2016). Out of six patient image sets, five had regions that were classified as fibrotic. These five image-sets were further used for mesh creation.

### 2.3 Geometrical mesh construction

In order to construct the 3D meshes we used a previously implemented automatic pipeline (Marciniak et al., 2016; Marciniak, 2017). First, slices were aligned and transformed to remove motion artifacts. Surfaces were then created and smoothed using Visualization Toolkit (VTK) (Schroeder et al., 1998). Finally, finite element 3D meshes, each consisting of ∼ 10 million elements with a mean resolution of 530 *μm*, were generated from the surfaces using gmsh (Geuzaine and Remacle, 2009). After mesh creation, we assigned myocardial fiber orientations using a Laplace–Dirichlet Rule-Based algorithm (Bayer et al., 2012). The five geometrical models, with fibrotic regions mapped onto the mesh, are shown in Supplementary Figure S1 in Supplementary Material. These meshes were further used to create two types of models with different fibrotic representation: cleft and non-cleft models.

### 2.4 Cleft models

We created three populations of cleft models. In the first, fibrosis was represented with clefts, decreased conductivity and ionic remodeling (clefts_*gm*_). The second population had clefts and ionic remodeling (clefts_*m*_). In the third, fibrosis was represented with clefts only (clefts_*only*_). Each cleft population consisted of 10 sets of models with different cleft densities as in Balaban et al. (2020), where the lowest cleft density was defined as zero clefts. In addition, we created one set of models with decreased conductivity as before, with zero cleft density and without ionic remodeling (clefts_*g*_). A summary of all parameter combinations is given in Table 1.

**TABLE 1:** Summary of all models and parameters. For cleft models, we varied *fib*_max_ in steps of 1, from 0 to 9. For non-cleft models, we varied the lower LGE threshold for core regions between 25, 50 or 75% below maximum LGE intensity. Values for conductivity and conduction velocity are listed in Table 1 in Supplementary Material. Ionic remodeling was based on measurements in hypertrophic cardiomyopathy and applied to the entire fibrotic region. Cleft_*g*_ models were only simulated as an illustrative case, to compare with the *fib*_max_ 0 case in cleft_*gm*_. Table cells with non-applicable information are marked by hyphen (−).

Model	*fib*_max_	Core size	Ionic remodeling	Conductivity changes
Cleft_*gm*_	0–9	—	Yes	Step-wise reduction
Cleft_*m*_	0–9	—	Yes	None
Cleft_*g*_	0	—	Yes	Step-wise reduction
Cleft_*only*_	0–9	—	No	None
Non-cleft_*c*_	—	25, 50 and 75	Yes	Slowly conducting core with border zone
Non-cleft_*nc*_	—	25, 50 and 75	Yes	Non-conducting core with border zone

#### 2.4.1 Cleft creation

**FIGURE 1:**
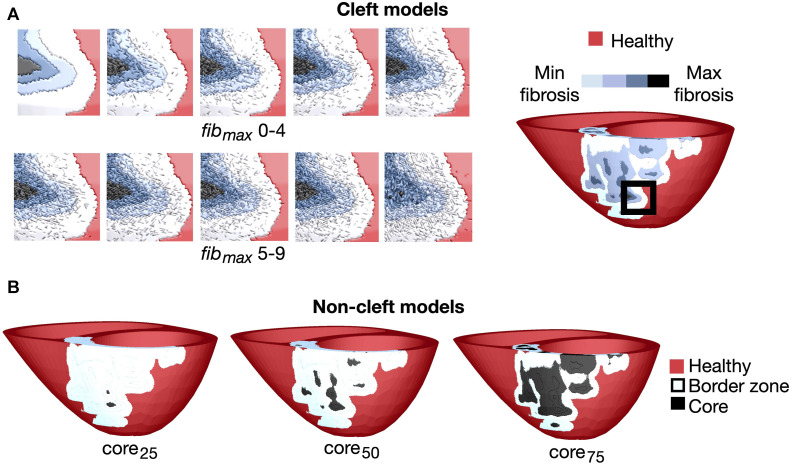
Overview of the fibrotic representations for patient 3. **(A)** Left: Cleft models with 10 levels of fibrotic cleft density (*fib*_max_ values of 0–9). Right: An example patient with *fib*_max_ = 0. The magnified region is indicated by the black box. **(B)**: Non-cleft models with core tissue and three different core sizes. The core region is defined as the top 25% (core_25_), 50% (core_50_) or 75% (core_75_) of the LGE intensity.

In cleft models, we represented fibrosis by using an existing method for mesh splitting (Balaban et al., 2020). The approach involves creating clefts in the mesh by disconnecting mesh elements to obtain local no-flux boundaries of current (Balaban et al., 2020). Since individual fibrotic clefts cannot be resolved with current LGE-CMR imaging technology, the method disconnects two neighbouring elements with a probability given by

$$p=p_{\max}\;|cos^{\alpha }\theta |I$$ (1)

where *p*_max_ scales the maximum cleft density, *α* determines the anisotropy, *I* is the normalised LGE intensity projected onto the mesh and *θ* is the angle between the element side and the fiber sheet normal direction (Balaban et al., 2020). In cases where an element is completely isolated from all its neighbours, it is removed from the mesh. All 10 levels of cleft density for an example patient are depicted in Figure 1A.

We sorted the parameter combination from minimum to maximum number of removed elements, and defined a corresponding indicator of global cleft density *fib*_max_, which takes on values from 0 to 9. The number of elements removed from each model for each parameter combination is given in Supplementary Figure S2 in Supplementary Material.

#### 2.4.2 Conductivities

In non-fibrotic tissue, we assigned conductivities tuned to match longitudinal and transverse conduction velocities of 54.4 and 33.5 cm/s, respectively, as done in previous studies (O’Hara et al., 2022).

For cleft models with reduced conductivity in fibrotic regions (clefts_*gm*_), we divided myocardial tissue into regions of 0%–25%, 25%–50%, 50%–75% and 75%–100% LGE-CMR intensity. A step-wise reduction in conductivity was used for all 10 cleft models, including in the control models without clefts (*fib*_max_ = 0). We decreased the conductivity in a similar way as in Balaban et al. (2020). The conduction velocity was reduced first in the transverse, then both myocardial directions, based on experimental recordings in patients with idiopathic dilated cardiomyopathy (Anderson et al., 1993). Specifically, in regions with image intensities 0%–25% and 25%–50%, longitudinal velocity was kept normal while transverse velocity was reduced with 40% and 80% respectively. For intensity ranges 50%–75% and 75%–100%, longitudinal velocity was reduced with 40% and 80% respectively and transverse velocity reduced by 80% compared to healthy tissue. The five tissue regions of differing conductivity (healthy tissue and four levels of fibrotic conductivities) in a cleft model of an example patient are depicted in Figure 1, most easily seen at top left (*fib*_max_ = 0).

Conduction velocities in both the healthy and fibrotic regions were obtained by tuning the conductivity values using a 10 cm long cable of myocardial tissue with 530 *μm* resolution. Conductivity values are listed in Supplementary Table S1 in Supplementary Material.

### 2.5 Non-cleft models

We created two sets of non-cleft models. One in which dense fibrosis was modelled as slowly conducting core tissue (non-cleft_*c*_) and one in which dense fibrosis was modelled as non-conducting core tissue (non-cleft_*nc*_). For both populations, we used three different ratios of core *versus* border zone. A summary of the parameter combinations is given in Table 1.

#### 2.5.1 Conductivities

As in the cleft models, we assigned conductivities based on the normalised LGE image intensities in fibrotic regions. From these intensities, we defined tissue as either belonging to the border zone or the fibrotic core. To investigate the effect of varying core size, we used three different lower thresholds of LGE intensity to define the core region: 25% (core_25_), 50% (core_50_) or 75% (core_75_) less than maximum LGE intensity, resulting in small, medium and maximum core extents respectively (Figure 1B). The remaining fibrotic regions were defined as border zone.

We used the same conductivities in healthy tissue as in the cleft models, corresponding to longitudinal and transverse conduction velocities of 54.4 and 33.5 cm/s, respectively. In the border zone, we tuned longitudinal and transverse conductivities to match values previously used to model NICM hearts: 43.2 and 17.9 cm/s, respectively (Shade et al., 2020; O’Hara et al., 2022).

In core tissue, we used different conductivities for the two sets of non-cleft models. In non-cleft_*nc*_ models, conduction block was achieved by setting the tissue conductivity of core regions to 10^–7^ S/m. In non-cleft_*c*_ models, we used a conductivity of 0.01 S/m based on experimental measurements of collagen (Bishop et al., 2010), representing dense interstitial fibrosis (Frangogiannis, 2021).

### 2.6 Cell membrane dynamics

In non-fibrotic tissue, for all models, we used the ten Tusscher model of the human ventricular cardiomyocyte (Ten Tusscher and Panfilov, 2006), with the late sodium current (*I*_*NaL*_) from the O’Hara-Rudy model (O’Hara et al., 2011) added to the total membrane currents.

**FIGURE 2:**
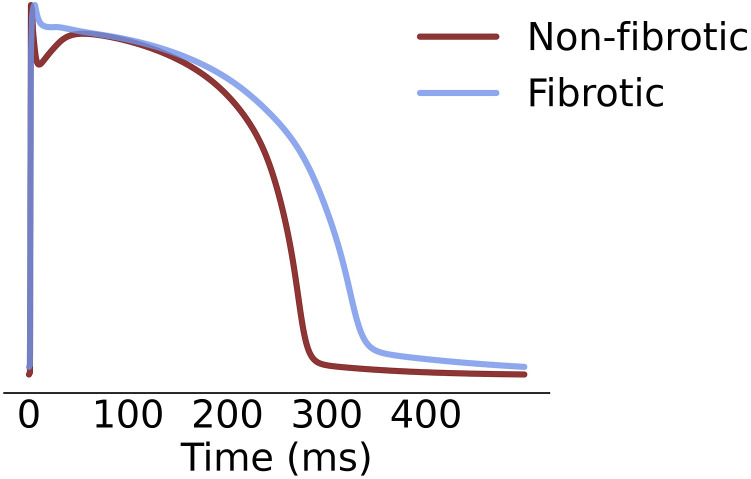
Hypertrophic cardiomyopathy (blue) and healthy (red) action potential.

For models with ionic remodeling in LGE regions (non-cleft, clefts_*gm*_ and clefts_*m*_), we incorporated the same ionic remodeling as in O’Hara et al. (2022). Due to a lack of reports on membrane dynamics in fibrotic regions of NICM patients, these modifications were based on measurements on cardiomyocytes from patients with hypertrophic cardiomyopathy (HCM) (Coppini et al., 2013). In this non-ischemic disease, fibrosis appears as a major alteration of the myocardium and is strongly associated with disease progression (Galati et al., 2016; Díez et al., 2020). The implemented ionic changes involved a 107% and 19% increase of the maximal *I*_*NaL*_ and *I*_*CaL*_ conductances respectively, a 34%, 27%, 85% and 15% reduction of *I*_*Kr*_, *I*_*Ks*_, *I*_*to*_ and *I*_*K*1_ respectively, a 34% increase of Na^+^/Ca^2+^ exchanger activity and a 43% reduction of Sarcoplasmic/Endoplasmic Reticulum Calcium ATPase activity. The resultant action potentials at 90% repolarization, after pre-pacing for 1,000 beats at 500 ms basic cycle length, were 280 and 330 ms in healthy and fibrotic tissue respectively (Figure 2).

### 2.7 Simulations

All electrophysiology simulations were run using the software openCARP (open Cardiac Arrhythmia Research Package (Plank et al., 2021)). Electrical propagation across tissue was simulated with the monodomain model (Keener and Sneyd, 1998). In order to determine whether reentrant arrhythmia could be induced, each model was paced from 17 sites on the left ventricle, one in each of the segments defined by the American Heart Association (AHA) (Cerqueira et al., 2002). We used a stimulation protocol previously described (Cheng et al., 2013; Arevalo et al., 2016; Maleckar et al., 2021). The automatic protocol consists of delivering a stimulation sequence (S1 - S4) to each site (see Supplementary Material for details). All pulses had 10 ms duration, 100 uA/cm2 current amplitude and 1 mm^3^ electrode volume. The simulation was continued for 4,000 ms after the last pacing or until all activation had ceased. In total, we conducted simulations in 37 models × 5 patients with LGE × 17 pacing sites, totaling 3,145 simulations.

### 2.8 Identification of reentry and reentry initiation sites

Simulated reentries and their initiation sites were identified automatically by analyzing activation patterns. Reentry was considered as any activation happening after the final pacing-induced activation of a specific vertex. That is, a reentrant wave was determined by noting the *n* + 1 activation of each vertex following the *n* successful pacing stimuli. Only reentrant activations were considered for further analysis.

Due to the cleft structure, some simulations had repeated activations in isolated vertices without forming a visible reentrant wave. These were likely caused by current propagating around a cleft, increasing the voltage in neighbouring, recently activated, vertices. In our analysis, activation sequences separated by <50 ms intervals were therefore interpreted as a single activation.

After selecting the activations caused by reentry, we used a graph-based approach to trace the reentry back to its initiation site(s) in time steps of 10 ms. An initiation cluster was defined as a collection of at least 1,000 connected vertices which were activated before all of its neighbouring vertices. We confined each cluster to the earliest time step at which it appeared. For the remaining analysis, we considered the geometrical center of the cluster as the initiation site. We further visually inspected all reentry simulations to confirm that the automatically detected initiation sites were consistent with our observations. The script for tracing the activation wave back to its source(s) is made available at https://github.com/lenamyk/reentry_simulations_post_processing.git. Technical details about the method are provided in Supplementary Material.

**FIGURE 3:**
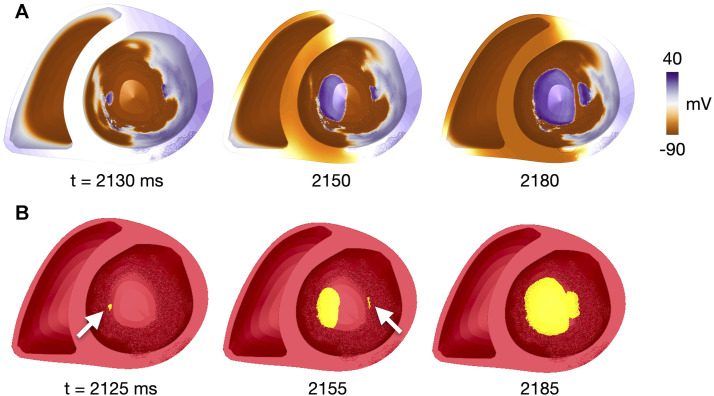
Identification of reentry initiation sites for patient 1, *fib*_max_ = 9, site 17. **(A)** Voltage map at time points close to those in **(B)**, given the temporal resolution of 10 ms for voltage recordings. **(B)** Clusters of connected vertices which have been reactivated after the last pacing-induced activation. Time steps are after the first reentry initiation (t = 2,125 ms, initiation site indicated by white arrow), after the second reentry initiation (t = 2,155 ms, initiation site indicated by white arrow) and after merging of the two reentrant waves (t = 2,185 ms). Time is given in ms after start of simulation. A movie of the simulated reentry is found in the Supplementary Video S1.

An illustrative example of the method, taken from a simulation with two reentry initiations, is shown in Figure 3. The first and second initiation cluster appeared at 2,125 and 2,155 ms after start of the simulation, respectively. The two initiation sites were defined as the centers of each cluster, indicated by the arrow. The reentries later merged to a single reentry, as shown at 2,185 ms. A movie of the reentry is given in the Supplementary Video S1.

### 2.9 Activation times

To illustrate the effect of increasing the fibrotic scaling factor on propagation, we calculated the time taken to activate the myocardium in six example cases. The examples considered were *fib*_max_ values 0, 5 and 9 and all three core sizes for patient 4. In all cases, time was measured from the first pacing stimulus until 95% of all vertices were activated. The 95% threshold was chosen to enable comparison between the models, since not all vertices in the cleft models were activated by pacing. In the models were all vertices were activated, we also calculated the time to 100% activation.

### 2.10 Reentry propagation patterns

**FIGURE 4:**
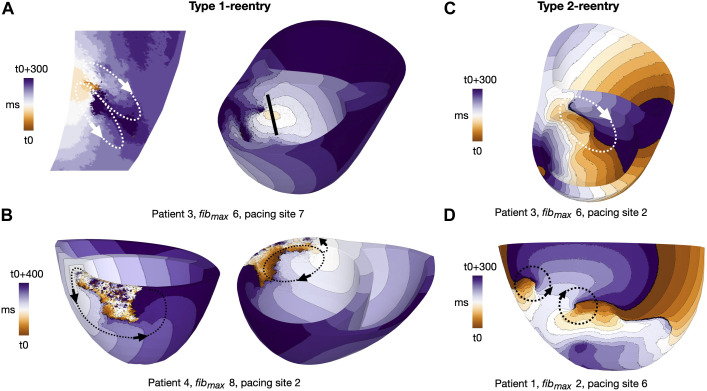
Activation maps for representative reentry patterns in clefts_*gm*_ models. Arrows indicate reentrant circuits. **(A)** Type 1-reentry with a small circuits in patient 3, *fib*_max_ = 6, paced from AHA segment 7. The figure shows a slice of the myocardial septum (left) where the reentry is initiated. The location of the slice is indicated by the black line (right). **(B)** Type 1-reentry with large circuits in patient 4, *fib*_max_ = 8, paced from AHA segment 2. **(C)** Single-loop type 2-reentry for patient 3, *fib*_max_ = 6, paced from AHA segment 2. **(D)** Double-loop type 2-reentry for patient 1, *fib*_max_ = 2, paced from AHA segment 6. Movies of Type 1- and Type 2-reentries in cleft models are given in the (Supplementary Videos S4, S5, respectively).

We classified reentries into one of two types based on visual inspection of propagation patterns. The first, Type 1, involved slow meandering through a narrow conducting channel which resulted in a double-loop reentry (Figures 4A, B). The second, Type 2, was a functional scroll wave reentry rotating close to an interface between healthy and fibrotic myocardium. These reentries involved single or multiple loops (Figures 4C, D). Example movies of the two reentry types are available in the Supplementary Videos S2−5.

### 2.11 Calculating LGE features per initiation site

To characterise the reentry initiation sites and investigate how these sites vary with parameter choice and morphology, we calculated selected LGE features surrounding each site. The patient-specific LGE intensities of fibrotic regions as segmented from the CMR images were first mapped onto each model. For each initiation site, we then calculated the mean and maximum LGE intensity as well as the total LGE volume within a 5 mm radius of the site. The radius was determined by calculating the distances between all vertices in the mesh and each initiation site. Vertices within a 5 mm distance and the elements belonging to these vertices were selected as the region of interest.

### 2.12 Segment-based analysis

Considering the strong association between LGE and arrhythmia in NICM patients (Theerasuwipakorn et al., 2023), we aimed to investigate how the number of reentry initiations varies with local, LGE-specific features. We thus divided the left ventricle into regions defined according to the 17 left ventricular AHA segments. Due to the limited number of reentries with a Type 2 morphology (35 initiation sites), we considered only Type 1-reentries (616 initiation sites). Using a negative binomial regression model, we tested the association between the number of reentries initiated in a segment, and selected features (LGE volume, LGE intensity and core volume). Effects of patient geometry, fibrotic scaling parameter (*fib*_max_ or core size) and AHA segment were accounted for by including these variables as random effects. The threshold for significance was taken at p < 0.05. The resulting models are listed in Supplementary Tables S2, S3 in Supplementary Material.

## 3 Results

### 3.1 Models with clefts, reduced conductivity and ionic remodeling

#### 3.1.1 Effect of cleft density on tissue activation

**FIGURE 6:**
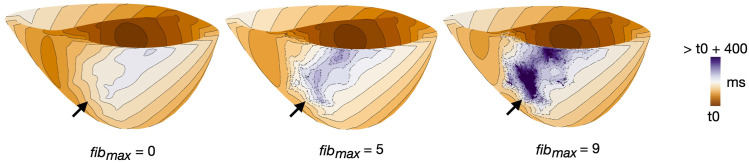
Example of differences in activation pattern for different cleft densities (*fib*_max_ = 0, 5 and 9) in clefts_*gm*_ models. Activation maps are for patient 3, during S1, paced from AHA segment 2. Arrows mark the regions of fibrosis.

Altering *fib*_max_ altered tissue activation in all cleft models (clefts_*gm*_, clefts_*m*_ and clefts_*only*_). The activation maps in Figure 6 illustrate differences in activation patterns for clefts_*gm*_ models of patient 3. For *fib*_max_ 0 (left), the first stimulus when paced from AHA segment number 2 resulted in smooth propagation throughout the myocardium. As *fib*_max_ was increased to 5 (middle), the wavefront within the fibrotic region became slower and more disordered. At *fib*_max_ 9 (right), the clefts cause considerable local conduction slowing and fragmentation.

For each of the three examples, we measured the time taken from the first pacing stimulus until global activation. For *fib*_max_ 0, 5 and 9, 95% of the myocardium was activated after 183, 239 and 309 ms, respectively. At *fib*_max_ 0, 100% of the myocardium was activated after 225 ms. For *fib*_max_ 5 and 9, multiple vertices remained inactivated throughout the pacing.

#### 3.1.2 Reentry sustainability, number of initiation sites and required number of extra stimuli

**FIGURE 7:**
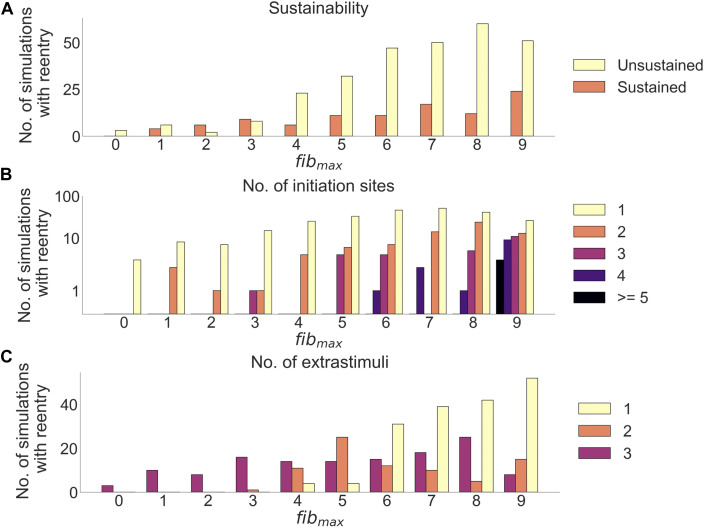
Effects of cleft density on reentry in clefts_*gm*_ models. **(A)** Number of sustained *versus* unsustained reentries. **(B)** Number of initiation sites (separate areas from which a reentry is formed), per simulation with reentry. **(C)** Number of extrastimuli delivered before reentry is initiated.

After delivery of arrhythmia induction protocol in clefts_*gm*_ models, reentry was observed in 382 out of 850 simulations (45%). Adjusting *fib*_max_ from 0 to 9 increased the proportion of simulations with reentry from 3.5% (3/85) to 88% (75/85). We measured how *fib*_max_ affected reentry sustainability, the number of initiation sites, and the initiation time (Figure 7).

Out of all simulations with reentry, 282/382 (74%) had unsustained ( < 4,000 ms) while 100/382 (26%) had sustained ( > 4,000 ms) reentry (Figure 7A). While for the lower levels of *fib*_max_ (0–3) there was only 38 reentries in total, there was a relatively high proportion of sustained reentries (mean: 42.0%), but with a large variation (standard deviation: 27.3%). When we increased *fib*_max_ to levels 4–9, we observed a considerably higher number of reentries (344 simulations with reentry in total). These simulations had a lower proportion of sustained reentries (mean: 21.5%) with a smaller variation (standard deviation: 3.5%). In most simulations, reentries originated from a single initiation site. However, as we increased *fib*_max_, the number of initiation sites increased (Figure 7B). For fibrotic levels of *fib*_max_ > = 6, we found reentry initiation from up to four separate locations in a simulation. Only for the highest fibrotic level, *fib*_max_ = 9, did we identify more than five initiation sites, with up to 11 initiation sites per reentry simulation.

Increasing *fib*_max_ lead to fewer extra stimuli needed to induce reentry (Figure 7C): For *fib*_max_ values 0–2, all reentries needed three premature extra stimuli to form (21 simulations). At *fib*_max_ = 3, 1/17 reentries were induced after two premature extra stimuli, while the remaining 16/17 (94%) reentries needed three premature extra stimuli to form. When increasing *fib*_max_ to 4, we were able to induce reentries using only a single extra stimulus. The number of reentries induced after one extra stimulus increased from 4/29 (14%) at *fib*_max_ = 4 to 52/75 (69%) at *fib*_max_ = 9.

#### 3.1.3 Reentry propagation patterns

We observed two distinct reentry morphologies. Most reentries were of Type 1 (*n* = 352 simulations, 92.1% of all simulations with reentry), previously defined as slowly propagating through narrow channels in fibrotic regions (Methods 2.10). The reentrant pathways in clefts_*gm*_ models were constricted by a combination of clefts and reduced conductivity. A spectrum of Type 1-reentry patterns was observed. Some were micro-circuits within the myocardium, appearing like focal activity when visualized on the endocardial surface (Figure 4A). Others followed circuits across relatively large sections of myocardium (Figure 4B). The Type 2-reentries - defined as functional rotors close to fibrotic-healthy interfaces (Methods 2.10, Figures 4C, D) - only made up a small subset of the reentries (n = 30 simulations, 7.9% of all simulations with reentry).

When separating reentries by morphology, a higher proportion of Type 1-reentries were unsustained compared to Type 2-reentries (78/352 (22.2%) vs 22/30 (73.3%)). On average, both reentry morphologies had between one and two initiation sites per simulation with reentry (1.75 ± 1.53 Type 1 vs 1.17 ± 0.37 Type 2). While Type 1-reentries were formed after one, two or three extra stimuli, all 30 Type 2-reentries required three extra stimuli to form.

#### 3.1.4 Effect of cleft density on initiation sites

**FIGURE 8:**
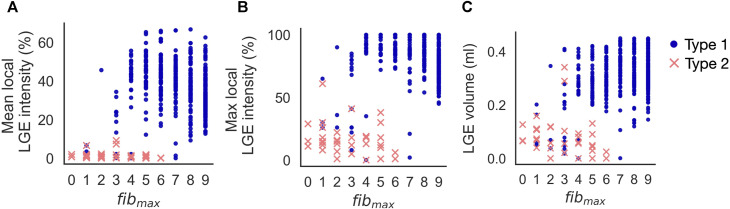
Increasing *fib*_max_ in clefts_*gm*_ models from 0 to 9 resulted in reentry morphology changing from 100% Type 2–reentry to 100% Type 1–reentry. Type 1-reentries are initiated in areas of higher mean LGE intensity **(A)**, higher maximum LGE intensity **(B)** and higher LGE volume **(C)** than Type 2-reentries.

Increasing *fib*_max_ from 0 to 9 in clefts_*gm*_ models resulted in reentry morphology changing from 100% Type 2-reentry to 100% Type 1-reentry. Initiation sites for the two reentry morphologies were characterised by different local LGE properties for varying cleft densities. Figures 8A, B shows the mean and maximum LGE intensities measured from patient CMR within a 5 mm radius of each initiation site (Method 2.11). Intensities were normalised so that 0% represents non-fibrotic tissue and 100% is the highest intensity for the patient. Consistent with our classification of reentry type, we observed two clusters of reentry initiation sites. Type 1-reentries were typically initiated in regions with a mean LGE intensity which was higher than the average intensity of all fibrotic regions (36.5% ± 12.29% vs average 24.98% ± 10.97%, Figure 8A). These sites also had high maximum LGE intensities (82.37% ± 16.22%, Figure 8B). Type 2-reentries were initiated at sites with low LGE (mean: 1.49% ± 2.04%, max: 17.57% ± 12.91%). The mean intensities for these sites only varied between 0.0% and 9.21% (Figure 8A). As we increased *fib*_max_, more reentries were initiated in higher LGE regions as the reentry morphology changed from Type 2 to Type 1. The LGE volume within a 5 mm radius of each initiation site is shown in Figure 8C. Type 1-reentries were initiated in areas with a larger extent of LGE than Type 2-reentries (0.31 ± 0.08 mL vs 0.08 ± 0.07 mL). Correspondingly, as we increased *fib*_max_ and the reentry morphology changed from Type 2 to 1, reentries were initiated in areas with higher LGE volume.

#### 3.1.5 Association between local LGE intensity and initiation sites

In the previous section, we looked at how reentry initiation varied with *fib*_max_. For this, we calculated LGE properties only around initiation sites. To measure the association between region-specific LGE features and reentry initiation, we must take into account all fibrotic regions, also those without reentry initiation. We therefore investigated how the number of reentry initiations vary for different ventricular AHA segments. Only Type 1-reentries, which had a high number of initiation sites (*n* = 616), were considered. Reentries were only initiated in segments with LGE. Furthermore, when accounting for LGE volume, the number of reentry initiations in a segment was significantly associated with the segment-specific LGE intensity (mean LGE: p < 0.001, maximum LGE: p < 0.001, negative binomial regression). Estimates for the regression models are presented in Supplementary Table S2 in Supplementary Material.

**FIGURE 9:**
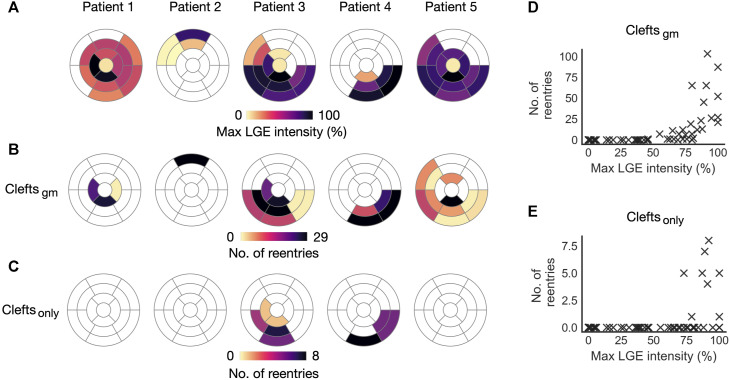
Initiation sites for Type 1-reentry in cleft models. Numbers of initiated reentries are aggregated over all parameter combinations (*fib*_max_0-9). **(A)** Maximum LGE intensity per segment projected from the patient CMR onto the five ventricular geometries. **(B, C)** Number of Type 1-reentries initiated in each segment for clefts_*gm*_ and clefts_*only*_ models. **(D, E)** Maximum LGE intensity per segment *versus* total number of reentries initiated in the segment for clefts_*gm*_ and for clefts_*only*_ models.

The association between LGE intensity and number of initiation sites is illustrated in Figure 9. Figure 9A shows the maximum LGE intensity for each segment in a patient. Figure 9B shows the total number of Type 1-reentries initiated per segment in clefts_*gm*_ models. There is a clear visual overlap between segments with high maximum LGE intensity and high numbers of reentry initiation. Conversely, segments with low LGE intensities had fewer or no reentry initiation. The number of reentry initiations *versus* maximum LGE per segment is presented in Figure 9D. We see that in segments where all tissue has < 50%, LGE intensity, no Type 1-reentries were initiated. From this point, the number of initiations increased with maximum LGE. For segments with max LGE intensity above 80%, there was at least one reentry initiation. The segment-based number of reentry initiations for each separate level of *fib*_max_ is given in Supplementary Figure S3 in Supplementary Material.

### 3.2 Models with only clefts

In the previous sections, fibrosis was modeled using clefts, ionic remodeling and reduced conductivity. We next investigated the effect of representing fibrosis using clefts alone. For clefts_*only*_ models, the resulting number of reentries per segment is presented in Figure 9C. These models were considerably less inducible; we observed a total of 27 simulations with reentry, only in patient 3 and 4 and only with the maximum level of clefts (*fib*_max_ = 9). The reentries were all of Type 1-morphology. Only 8 segments had reentry initiation, all of which were overlapping with segments of reentry initiation in clefts_*gm*_ models. Due to the small sample size, we did not analyze the association between LGE features and number of reentries. However, all 8 segments with reentry initiation had high values of maximum LGE intensity ( >73% , Figure 9E), consistent with the Type 1-reentries in clefts_*gm*_ models.

### 3.3 Ionic remodeling in fibrotic regions

We incorporated remodeled membrane dynamics in fibrotic regions of clefts_*gm*_ models as detailed in Methods 2.6. Close to the transitions between fibrotic and healthy regions, we observed initiation of Type 2-reentries. These were formed when a temporary conduction block prevented propagation from one type of tissue to another, causing parts of the activation wave to spiral around the tissue interface. In contrast, we did not observe this morphology in models without ionic remodeling (clefts_*only*_).

**FIGURE 10:**
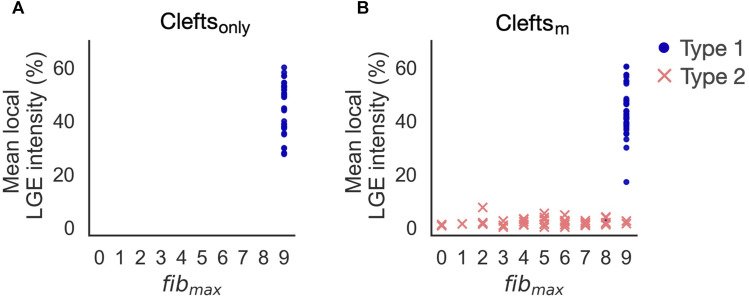
Reentry initiation in clefts_*only*_ **(A)** and clefts_*m*_ models **(B)**. Adding ionic remodeling to fibrotic regions gives rise to reentries in low LGE areas (Type 2). Type 1-reentries are only initiated for the highest levels of cleft density (*fib*_max_ = 8 or 9).

To investigate if Type 2-reentries were caused by ionic remodeling or conductivity reduction, we created a set of cleft models with ionic remodeling but normal conductivity (clefts_*m*_). Compared with clefts_*only*_, the addition of ionic remodeling had little effect on Type 1-reentry, whereas the number of Type 2-reentries increased from 0 to 42 initiations (Figure 10). This is even higher than the number of Type 2-reentries previously found in clefts_*gm*_ models (35 initiations). In both clefts_*gm*_ and clefts_*m*_ models, Type 2-reentries were observed for *fib*_max_ = 0, showing that ionic remodeling were sufficient for Type 2 initiation. Additionally, we created a set of models with *fib*_max_ = 0 with reduced conductivity but no ionic remodeling in fibrotic regions. We observed no reentry in these models. Together, these results demonstrate that ionic remodeling was key to Type 2-reentry initiation in our simulations.

### 3.4 Non-cleft models

We first created a set of models with non-conducting core tissue surrounded by a border zone with reduced conductivity (non-cleft_*nc*_). In these, reentry was induced in only 9 out of 255 (3.5%) simulations. All reentries were of Type 2, initiated at the borders between healthy and fibrotic tissue (see Supplementary Figure S4). In a subsequent set of models (non-clefts_*c*_ models), we represented core tissue using very low conductivity (0.01 S/m), which made the models more inducible to reentry.

#### 3.4.1 Effect of core size on tissue activation

**FIGURE 11:**
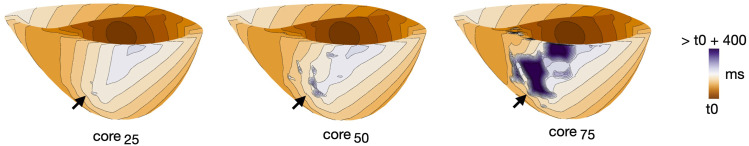
Example of differences in activation pattern for different core sizes (core_25_, core_50_ and core_75_) in non-clefts_*gm*_ models. Activation maps are all for patient 3, during S1, paced from AHA segment 2. Arrows mark the regions of fibrosis.

While for cleft models we looked at effects of varying *fib*_max_, in non-clefts_*c*_ models we looked at effects of varying core size. Figure 11 illustrates differences in activation patterns for non-clefts_*c*_ models of patient 3. When pacing from AHA segment number 2 and looking at activation after the first stimulus, the smallest core size (Figure 11, left), had the most continuous wavefront. As we increased the core size to core_50_ (Figure 11, middle), we observed multiple distinct islands of delayed activation within the fibrosis. The largest core size (Figure 11, right) had large regions of severe conduction slowing.

For the three models in Figure 11, we measured the time taken from the first pacing stimulus until the entire tissue was activated. For core sizes 20, 50 and 75, it took 183, 187 and 244 ms, respectively, to activate 95% of the myocardium by pacing. For 100% activation, it took 229, 303 and 513 ms, respectively.

#### 3.4.2 Reentry inducibility, number of initiation sites and required number of extra stimuli

**FIGURE 12:**
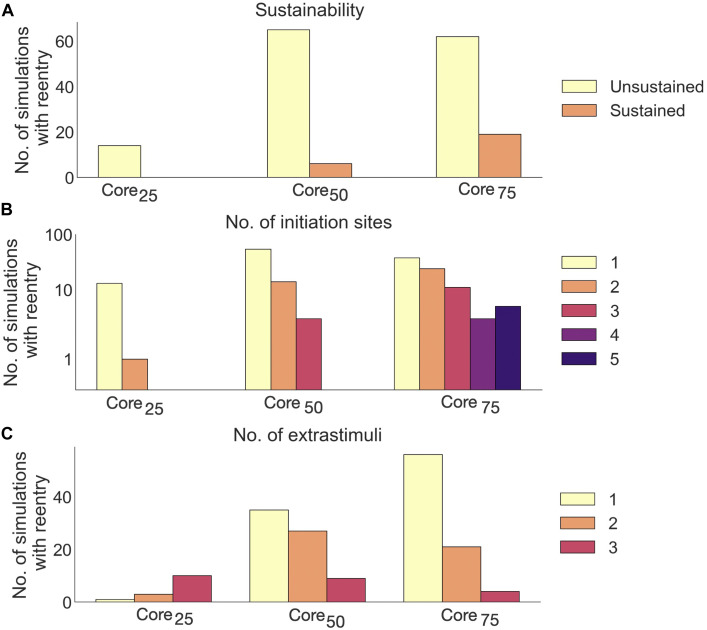
Effects of core size on reentry in non-clefts_*c*_ models. **(A)** Number of sustained *versus* unsustained reentries. **(B)** Number of initiation sites (separate areas from which a reentry is formed), per simulation with reentry. **(C)** Number of extrastimuli delivered before reentry is initiated.

Extending the core size from core_25_ to core_75_ increased the proportion of simulations with reentry from 14/85 (16.5%) to 81/85 (95.3%). Out of these reentries, only 25/166 (15%) were sustained. The proportion of sustained reentries ( > 4,000 ms) increased with core size, from 0/14 (0%) in core_25_ to 19/81 (23.5%) in core_75_ (Figure 12A).

A majority of reentries (105/166, 63.3%) were initiated from a single site (Figure 12B). The number of initiation sites also increased with core size: In core_25_, reentries had only one or two initiation sites. Extending the core size to core_50_ resulted in reentries with up to three initiation sites. For core_75_, we observed up to 5 initiation sites per simulation.

Fewer extra stimuli were required to induce reentry in models with large compared to small core sizes: The number of reentries induced after a single extra stimulus increased from 1/14 (7%) in core_25_ to 56/81 (69%) in core_75_.

#### 3.4.3 Reentry propagation patterns

**FIGURE 5:**
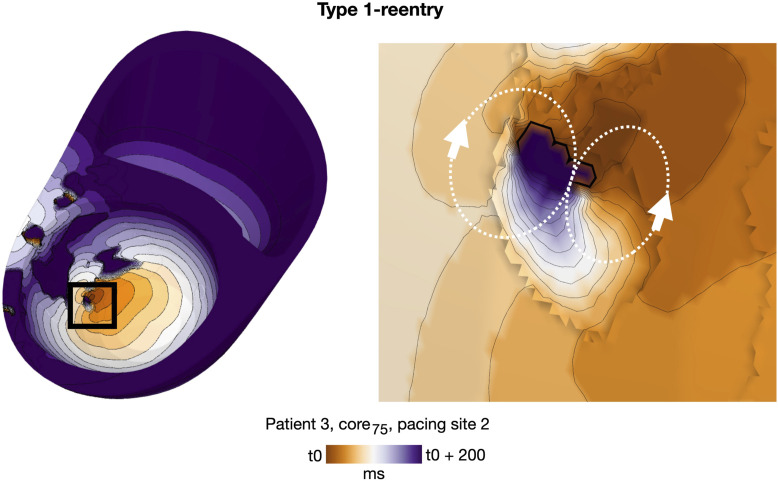
Representative example of a double-loop Type 1-reentry in a non-cleft model with slowly conducting core tissue (patient 3, core_75_, paced from AHA segment 2). The region of reentry initiation (black box, left) is magnified (right). A movie of the reentry is given in the Supplementary Video S2.

As in cleft models, we observed both Type 1 and 2 reentry patterns (Methods 2.10). The vast majority, however, were Type 1-reentries (n = 162 simulations, 97.6% of all simulations with reentry). Differing from cleft models, the conduction slowing which gave rise to Type 1-reentry in non-cleft models was entirely caused by the reduced conductivity. The propagation slowing allowed the surrounding myocardium to repolarize whilst leaving isolated islands of activation. The activation wave could then escape into surrounding myocardium and create reentry (Figure 5). Type 2-reentries, with morphology described previously (Figures 4C, D), only appeared in four simulations (2.4% of all simulations with reentry). In all four simulations, these required three extra stimuli to form, were initiated from either one or two sites and were unsustained.

#### 3.4.4 Effect of core-to-border ratio on initiation sites

**FIGURE 13:**
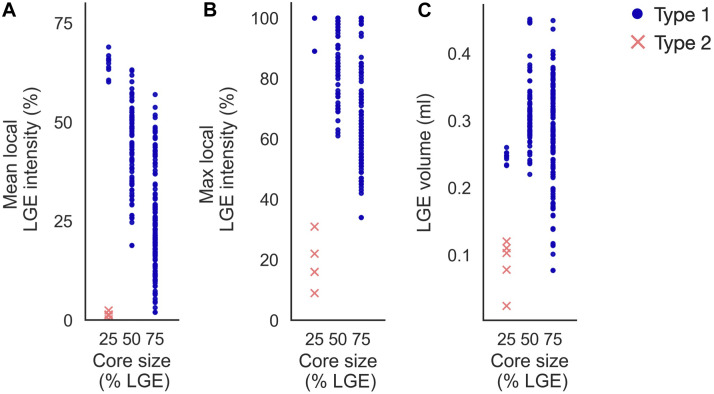
Initiation sites in non-cleft models with slowly conducting core tissue (non-cleft_*c*_). Type 1-reentries are generally initiated in areas of higher mean LGE intensity **(A)**, higher maximum LGE intensity **(B)** and higher LGE volume **(C)** than Type 2-reentries. For the smallest core size (core_25_), Type 1-reentries are confined to regions of high LGE intensity. When core sizes are increased, Type 1-reentries are initiated from a wider range of LGE intensities. Core sizes 25, 50 and 75 represent core_25_, core_50_ and core_75_ respectively.

Increasing core size affected reentry incidence and morphology in non-cleft_*c*_ models. The number of reentry initiations increased from 15 to 156 when increasing the core extent from core_25_ to core_75_. Figure 13 shows the LGE intensity and LGE volume within a 5 mm radius surrounding each initiation site. As in the cleft models, Type 1-reentries were initiated in areas with higher LGE intensity (mean: 32.65% ± 15.86%, max: 75.51% ± 18.28%) than Type 2-reentries (mean: 1.29% ± 0.72%, max: 18.8% ± 7.36%, Figures 13A, B). Type 1-reentries were also initiated in areas with higher LGE volume than Type 2 (0.29 ± 0.07 mL vs 0.09 ± 0.03 mL, Figure 13C).

Increasing the core size changed the morphology from both reentry types to only Type 1. The extent of core tissue also determined the area from which Type 1-reentries can be initiated. Hence, increasing the core size resulted in more Type 1-reentry initiation in lower LGE areas and fewer initiations in higher LGE areas (typically deeper within the core tissue). For example, at core_25_, all Type 1-reentries were initiated within 60%–69% mean intensity on CMR. This range was lowered to 19%–63% for core_50_, and even further to 2%–57% in core_75_.

#### 3.4.5 Association between local core volume and initiation sites

**FIGURE 14:**
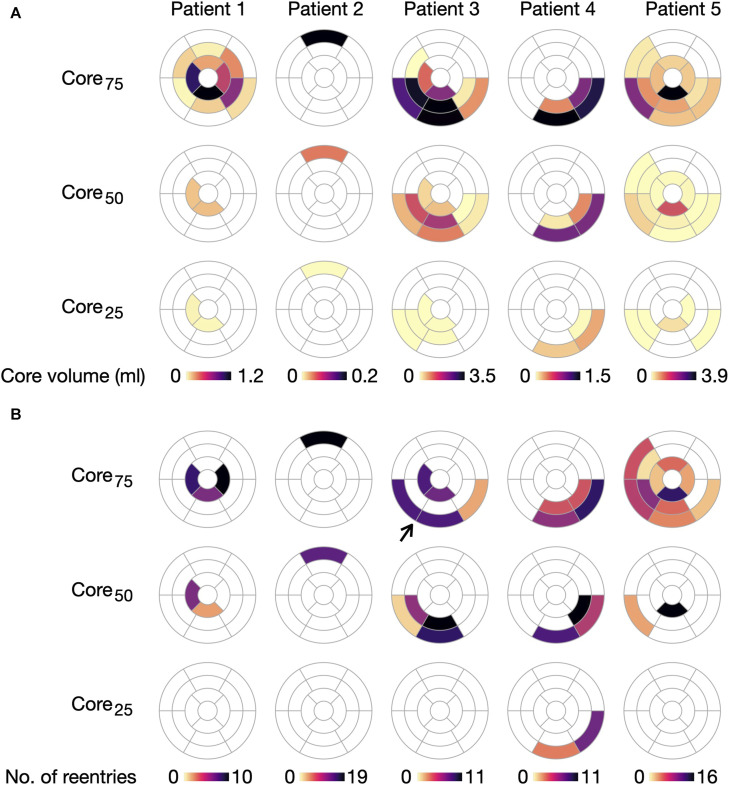
Initiation of Type 1-reentries is associated with core size in non-cleft_*c*_ models. **(A)** Core volume per AHA segment projected for the five ventricular geometries. **(B)** Number of Type 1-reentries initiated in each segment for each model. The number of reentries increases when extending the core size from core_25_ to core_75_. Arrow marks a case where two segments with dense fibrosis have no reentry initiation. For this case, reentry is instead initiated in the surrounding segments.

As for cleft models, we performed a segment-based analysis to investigate the association between local fibrosis intensity and reentry initiation. We again considered only Type 1, of which there were a relatively high number of reentries initiated (*n* = 162). For a segment with a fixed LGE volume, the number of initiations was correlated with the amount of core tissue (p <0.018 , negative binomial regression). This correlation is illustrated in Figure 14: The core volume in each segment of each model (Figure 14A) overlaps with the number of Type 1-reentries initiated in the segment (Figure 14B). Across all models, reentries were only initiated in segments with at least 0.1 mL core tissue (55/138, 39.9% of all segments with LGE). For segments with core volumes above 0.7 mL (22/138 15.9% of all segments with LGE), almost all had reentry initiation. The exceptions were two segments in the center of a large fibrotic region (see arrow in Figure 14B, patient 3, core_75_). In these, reentries were instead initiated from the surrounding layers of core tissue.

With respect to model parameters, the number of segments with reentry initiation increases as the core volume is extended from core_25_ to core_75_. For example, only two segments in patient 4 have Type 1-reentry for core_25_, compared to 24 segments across all patients for core_75_. We refer to Supplementary Figure S5 in Supplementary Material for an overview of segments with Type 2-reentries.

## 4 Discussion

The key findings of this study are:

• We observed a strong association between LGE intensity and the location of Type 1-reentries in both cleft and non-cleft models. This is because these reentries were linked to conduction slowing, either due to clefts or reduced tissue conductivity. As a result, both modeling approaches gave rise to similar reentry locations.
• Changing the fibrotic scaling parameter leads to a change in reentry morphology and location, suggesting the need to tune this parameters to the patient or the disease.
• Ionic remodeling in fibrotic regions gave rise to rotors initiated close to fibrotic-healthy interfaces (Type 2-reentries). These reentries were likely the result of spatial transitions in membrane dynamics.
• The cleft models in our study had a higher variety of circuit sizes and patterns than non-cleft models. This might be because clefts assigned proportionally to LGE intensity result in a higher degree of local differences in conduction velocity than in models with only two levels of fibrosis (core and border zone).

Each point is discussed in further detail accordingly in the following four sections.

### 4.1 Fibrotic density and location of reentry sites

Multiple studies have identified reentry as an arrhythmic mechanism in NICM patients (Sasaki et al., 2013; Shirai et al., 2019; Nishimura et al., 2020; Tung et al., 2020; Nishimura et al., 2021a). Finding the location of a reentrant circuit in a patient is critical for targeting ablation and may provide insight into the reentrant mechanism. We therefore identified the initiation sites - a key part of the reentrant circuit - for all reentries in our simulations. We did not map the circuits throughout the entire reentry; however, in most of our simulations (569/573, 99.3% of Type 1-reentries and 45/74, 60.1% of Type 2-reentries), the reentrant circuits did not meander throughout the myocardium, but remained stable and close to the initiation site.

We only observed reentry initiation in segments with fibrosis. However, the location within these segments varied depending on modeling choices. For fibrosis modelled as conductivity reduction (non-clefts_*c*_) or both clefts and conductivity reduction (clefts_*gm*_), there was a significant association between reentry initiation and local intensity of fibrosis; the higher the LGE intensity in a segment, the more reentries were initiated in that area. Such a correlation between LGE intensity and number of reentries have also been found in other cardiac models with fibrosis (Roney et al., 2016). In the present study, incorporating ionic remodeling in fibrotic regions resulted in Type 2-reentries forming at transitions into these regions. In consequence, the locations of Type 2-reentries strongly depends on the choice of intensity threshold used to define the fibrotic regions.

Experimental studies on NICM patients have investigated the relation between fibrotic density and different components of the reentry circuit: the entrance and exit to a central common pathway (isthmus), the isthmus itself, and the outer loop of the reentry. Nishimura et al. (2021a) found that the ventricular reentry circuits propagated through tissue ranging from dense fibrosis to normal myocardium. The median proportions of dense scar among the NICM patients were highest at the entrance (61%) and lowest (31%) at the outer loop. The overlap of reentry circuits with regions of dense fibrosis, in addition to the large variation of fibrotic densities throughout the circuit is more consistent with the Type 1-reentries than the Type 2-reentries in our study. Consistent with the Type 1-reentry in our study, Piers et al. (2014) found that the LGE intensities at isthmus or exit sites were higher than the average scar intensity in NICM patients. These sites were, however, also typically located within 5 mm of the core-border zone transition (defined at 50% LGE intensity), from which they argued that this intermediate intensity represents a critical substrate for reentry in NICM patients. Consistent with these findings, Fernández-Armenta et al. (2012) reported that arrhythmic risk in dilated cardiomyopathy patients increased when a higher percentage of the fibrotic region was border zone compared to core. Similarly, Leyva et al. (2022) found the extent of border zone to be a better risk predictor than total fibrotic extent in a cohort of ICM (ischemic cardiomyopathy) and NICM patients.

### 4.2 Fibrosis density scaling parameter

A challenge in LGE-based cardiac modeling is that the relative image intensities are mapped to some absolute level of fibrosis. The modeler therefore has to choose the value of parameters which specify the fibrotic density. This parameter choice may, however, affect both inducibility and morphology of reentries. To investigate these effects, we varied a fibrotic scaling parameter for each model type: the overall cleft density (*fib*_max_ 0–9) in cleft models and core size (core_25/50/75_) in non-cleft models.

Increasing the fibrotic scaling parameter lead to increased reentry inducibility, consistent with previous simulation studies (Balaban et al., 2020; Balaban et al. 2018). For high scaling parameters, Type 1-reentries became the sole reentry mechanism. This may be because Type 2-reentries were less inducible, requiring a higher number of extrastimuli to form than Type 1. Since no more extrastimuli were delivered once reentry was detected, the formation of Type 1-reentry prevented Type-2 initiation during the same simulation. Hence, a higher number of Type 1-reentry caused by increased fibrotic density leads to fewer observed Type 2-reentries.

In models with high fibrotic scaling parameters in our study, the fibrotic density within each model ranged from non-fibrotic (in non-LGE areas) to complete conduction block (in the highest LGE areas). Any potential reentry will therefore be initiated in some intermediate LGE range, consistent with reports of reentry location in NICM patients (see Section 4.1). Of further note is that, when increasing the fibrotic scaling factor in our cleft_*gm*_ models, the global cleft density increases, while the membrane dynamics and conductivity values remains the same. Since clefts were assigned proportionally to this intensity in a smooth gradient fashion, whilst membrane and conductivity values were categorised into only a few different discrete values, an increase in cleft density also increased the degree to which local variations in LGE intensity were represented.

It should be taken into account, however, that fibrosis in NICM patients is highly varied and often diffuse, as opposed to the compact fibrosis seen in ICM (Glashan et al., 2018). For patients where the highest LGE intensities correspond to only mild fibrosis, as may be the case for some NICM patients, low scaling parameters may be more appropriate. A recent NICM modelling study reports significant improvement in risk stratification when implementing patient-specific fibrosis thresholds on CMR by use of T1 mapping (O’Hara et al., 2022), suggesting a need for patient-specific fibrotic scaling parameters in NICM models.

### 4.3 Modeling spatial transitions between tissues

Previous studies have used spatial transitions in membrane dynamics between healthy and fibrotic tissue similar to those used here (O’Hara et al., 2022). We found that incorporating ionic remodeling in fibrotic regions gave rise to Type 2-reentries: rotors forming when the activation wave crossed the border between healthy and fibrotic tissue. However, sudden transitions in membrane dynamics may not represent realistic transitions cardiac tissue. Indeed, in the atrial study by Roney et al. (2016), models with step-wise changes in membrane dynamics and conductivity were worse at locating clinically observed rotors than models with only clefts or percolation assigned proportionally to the LGE intensities. Assigning changes in membrane dynamics proportionally to the LGE intensities may result in fewer Type 2-reentries close to fibrotic-healthy transitions than in our models.

Previous modeling studies have often represented fibrotic tissue in NICM and ICM patients as non-conducting core tissue surrounded by a border zone O’Hara et al., 2022; Shade et al., 2020; Cartoski et al., 2019; Arevalo et al., 2016). In NICM patients, a non-conducting core may not accurately represent fibrosis, as these patients could still have viable myocytes in regions assigned as core tissue. As an alternative, we created non-cleft_*c*_ models with severe conduction slowing in core tissue, forming a substrate for Type 1-reentries. However, representing entire regions with severe conduction slowing may conversely overestimate the likelihood of reentry. By modeling the degree of fibrosis as proportional to LGE intensity, the cleft models offers an intermediate alternative which accounts for nuances in intensity distribution obtained from CMR.

### 4.4 Reentrant propagation patterns

The propagation patterns of ventricular reentry circuits in NICM patients have previously been studied. Nishimura et al. (2021b) found both single and double loop reentry patterns in a cohort of NICM and ICM patients. In the present study, single loop reentries required transitions in membrane dynamics (Type 2-reentries), whereas double loop patterns were formed for both Type 1- and Type 2-reentries and were observed in all model types. Our results show that certain reentrant patterns, such as the single-loop scroll wave, may be more easily induced when incorporating ionic remodeling in fibrotic regions, as opposed to only clefts or conductivity changes.

Tung et al. (2020) found that reentries typically had long and narrow conducting channels in NICM patients. Such reentries might be modelled using a cleft representation of fibrosis, which constrains the propagation to specific pathways in the myocardium. Swarup et al. (2002) and Hsia et al. (2003) reported reentrant pathways across regions of low voltage tissue. In the present study, some of the Type 1-reentries in cleft models followed long conducting channels through the fibrotic region, consistent with these observations. The heterogeneous conduction properties introduced by the clefts resulted in a wide range of circuit sizes and propagation velocities. In contrast, all Type 1-reentries in non-cleft models presented as small, isolated islands of activation formed by the severe conduction slowing in core tissue. The low variety in reentry morphology for non-cleft models compared to cleft models is likely due to the categorisation of fibrosis into either core or border zone, constraining the conduction velocity to only three possible values. Although it is not yet clear whether the variation observed in cleft models represents that of the patients, these findings highlights the potential of cleft models to represent a spectrum of fibrotic morphologies.

Previous simulation studies in 2D (Balaban et al., 2018) and 3D (Balaban et al., 2020) have modeled fibrosis in NICM patients using clefts and reduced conductivity, but without ionic remodeling. In the latter study, all reentries involved meandering through convoluted pathways within the fibrosis. The preceding 2D study, however, reported both reentry circuits of varying sizes and rotors with a single organizing center. In the present study, reentry in models without ionic remodeling were all Type 1-reentries, consistent with Balaban et al. (2020). However, Type 1-reentries with wide conducting channels could potentially appear as having a single organizing center when viewed in a 2D short axis plane. Differences in reentry patterns may also be due to patient-specific differences in LGE distribution.

Recent work simulated reentry in NICM patients by representing fibrosis with conductivities and membrane dynamics that were similar to our non-cleft_*nc*_ models (O’Hara et al., 2022). In contrast to our non-cleft_*nc*_ population, however, some of their models had narrow paths through which reentry propagated. The intermingling between core and border regions can give rise to conducting channels for reentry (Andreu et al., 2011; Perez-David et al., 2011; Fernández-Armenta et al., 2013; Berruezo et al., 2015). Although such isthmuses may have been present in our patient cohort, the limited resolution of LGE images challenges translation of these structures onto the models in this study. Identifying these channels is further challenged by dependence on the threshold chosen for the separation between core and border zone. This highlights an advantage with the cleft population, which avoids the selection of such thresholds.

In addition to reentrant circuits, arrhythmia arising from focal activation has been reported in electrical mapping studies of NICM patients (Pogwizd et al., 1998; Anderson et al., 1993; Soejima et al., 2004). It has been proposed that focal activation reported in mapping studies may arise from micro-reentry, as the spacing between electrodes may not be small enough to detect the micro-reentrant circuit (Ideker et al., 2009). Many of the Type 1-reentries in our clefts and non-cleft models had micro-reentrant circuits that appeared similar to focal activity, propagating radially out from a single point. Other simulation studies have also demonstrated that micro-reentry can give rise to focal-like ectopic beats (Oliveira et al., 2018).

### 4.5 Limitations

Due to the difficulty of obtaining detailed LGE images of patients with specific pathologies, only five patient geometries were included in this study. This limits the variety in LGE configurations considered. Furthermore, since there is a lack of experimental data on membrane kinetics in NICM, we described fibrotic ionic changes by using a model of HCM electrophysiology, a disease which is typically associated with fibrosis. The previous use of these membrane dynamics for modeling reentry in HCM patients with both diffuse and focal fibrosis (O’Hara et al., 2022) further motivated our choice of investigating the impact of this modeling choice on reentry. While we observed micro-reentrant circuits which appeared similar to focal activation, arrhythmia due to genuine focal activity was considered outside the scope of this study and could be addressed by future work. Since it is not possible to investigate all potential fibrosis representations, our study is limited to a certain range of parameters. Exploring parameter combinations beyond this range may lead to different reentry dynamics. For example, certain thresholds of LGE intensity for defining core tissue could reveal reentrant pathways through border zone constrained by core tissue, a type of reentry which was not observed in this study. The resolution of the LGE images also limits the amount of information that can be represented with each modeling method. Our findings may therefore be affected by the resolution of the LGE image used in this study. Due to a lack of clinical measurements available for our patient cohort, it is unclear which of the models most accurately represented the arrhythmic mechanisms in this specific population. Future experimental studies could further investigate reentry patterns and the association between LGE intensity and reentry location in NICM. By providing measurements which can be compared with computational predictions, such studies would offer a substantial advancement towards determining the optimal method of modeling fibrosis in NICM.

### 4.6 Conclusion

We investigated how different fibrosis modeling choices affected arrhythmic mechanisms in personalised ventricular models of NICM patients. To this end, we compared two types of models. In one, fibrotic texture was incorporated as small non-conducting structures with a density proportional to LGE intensity (cleft models). In the second, fibrosis was divided into either core tissue or border zone, and represented solely as changes in conductivity and membrane dynamics (non-cleft models). The effect of parameter choice (fibrotic cleft density or extent of core tissue) on morphology as well as location of reentry initiation was investigated.

In both model populations, we observed two reentry morphologies. Type 1-reentries slowly meandered through fibrotic regions and included narrow channels of propagation. The initiation sites of these reentries were associated with the local intensity of fibrosis (LGE intensity or core size). Type 2-reentries were functional rotors appearing close to transitions between healthy and fibrotic tissue. Parameter changes which increased the fibrotic density (increased cleft density or core-to-border ratio) led to a change in reentry morphology from Type 2 to Type 1. Multiple similarities were observed between cleft and non-cleft models. The predominant reentry type was Type 1-reentry. Both clefts and reduced tissue conductivity causes local conduction slowing which may give rise to Type 1-reentries. In cleft and non-cleft populations, the number of Type 1-reentries as well as the location of their initiation sites were strongly linked to fibrotic density. Thus, cleft and non-cleft models often predicted similar reentry locations and morphology. In both populations, reentry morphology and location was largely determined by the fibrotic scaling parameter, highlighting the importance of parameter tuning when modeling reentry in NICM.

## Data Availability

The code used for identifying source(s) of activation from activation times on a computational mesh is available at https://github.com/lenamyk/reentry_simulations_post_processing.git.
