# Supplementary material for: Fibrosis modeling choice affects morphology of ventricular arrhythmia in non-ischemic cardiomyopathy
Source: Front Physiol. 2024 Mar 18;15:1370795. doi: 10.3389/fphys.2024.1370795 (PMC10986182; doi:10.3389/fphys.2024.1370795)
Supplement: Supplementary file 2 [file DataSheet1.PDF]

Supplementary Material

1 GEOMETRICAL MODELS

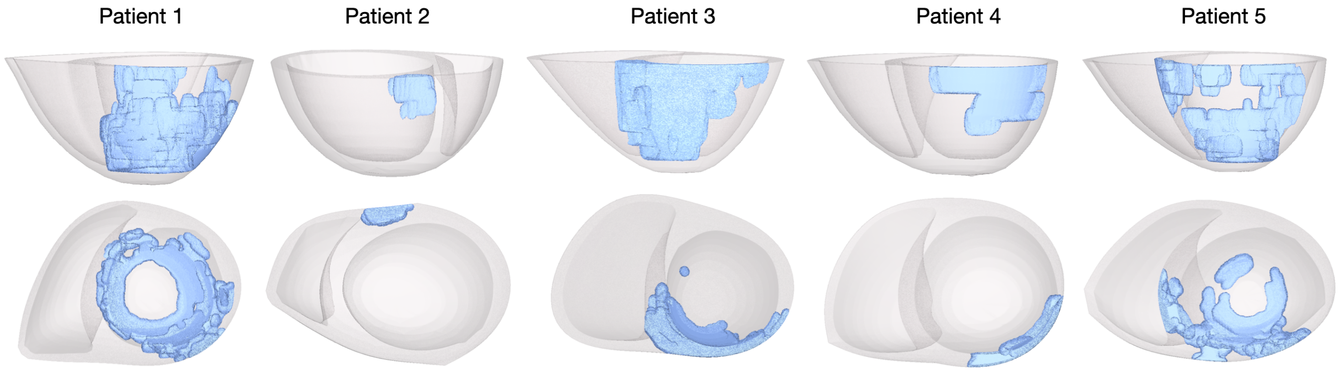

Figure S1: Geometrical models with regions of LGE (blue).

2 ELECTROPHYSIOLOGY

Cleft models with conductivity reduction and membrane changes

	g_{il}	g_{el}	g_{it}	g_{et}	g_{in}	g_{en}	CV_l	$CV_{t,n}$
Healthy	0.2564	0.9210	0.1353	0.4859	0.1353	0.4859	54.4	33.5
Minimum fibrosis	0.2564	0.9210	0.065	0.23	0.065	0.23	54.4	20.1
	0.2564	0.9210	0.02	0.07	0.02	0.07	54.4	6.7
	0.11	0.43	0.02	0.07	0.02	0.07	32.6	6.7
Maximum fibrosis	0.03	0.10	0.02	0.07	0.02	0.07	10.9	6.7
**Non-cleft models with conducting core**								
Healthy	0.2564	0.9210	0.1353	0.4859	0.1353	0.4859	54.4	33.5
Border zone	0.1883	0.6763	0.0622	0.2234	0.0622	0.2234	43.2	17.9
Core region	0.01	0.01	0.01	0.01	0.01	0.01	*	*
**Non-cleft models with non-conducting core**								
Healthy	0.2564	0.9210	0.1353	0.4859	0.1353	0.4859	54.4	33.5
Border zone	0.1883	0.6763	0.0622	0.2234	0.0622	0.2234	43.2	17.9
Core region	10^{-7}	10^{-7}	10^{-7}	10^{-7}	10^{-7}	10^{-7}	**	**

Table S1. Parameter values for conductivity (S/m) in the longitudinal, transverse and normal fiber direction. The tuned conduction velocities in the longitudinal (CV_l) and transverse and normal direction ($CV_{t,n}$) are given in cm/s. * Conductivity of 0.01 S/m resulted in failed propagation in a 1D rod, while in an example patient with the largest core size ($core_{75}$), the apparent conduction velocity measured from close to the core edge to the core center was 2.5 cm/s. ** Conductivity of 10^{-7} S/m resulted in conduction block in both a 1D rod and in all 3D meshes.

3 CLEFT PARAMETERS

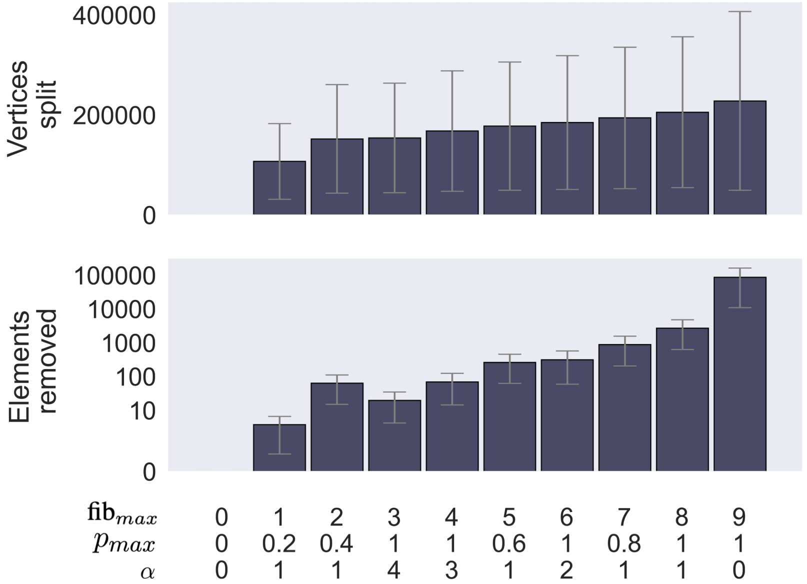

Figure S2: Number of vertices split **(A)** and elements removed **(B)** per model for each combination of cleft parameters. Clefts are created with a probability of $p = p_{max}\ |cos^{\alpha}\theta|\ I$, where p_{max} scales the maximum cleft density, α determines the anisotropy, I is the normalised LGE intensity projected onto the mesh and θ is the angle between the element side and the fiber sheet normal direction. Parameter combinations were sorted by number of vertices removed and assigned a value fib_{max}, used to denote cleft density level.

4 STIMULATION PROTOCOL

- **S1**: Each site is initially stimulated with five pulses, separated by 350 ms intervals.
- **S2**: An S2 stimulus follows 200 ms after the last S1 pulse. If the initial S2 stimulus fails to propagate, the interval is first increased in steps of 50 ms until the stimulus propagates. The S1-S2 interval is then reduced in steps of 10 ms until either reentry is induced or the stimuli fails to propagate.
- **S3**: Continuing from the last successful S2 stimulus, an S3 stimulus is delivered after 250 ms. The procedure of increasing or decreasing the stimulus interval is repeated for the S3 stimulus.
- **S4**: Finally, an S4 stimulus is delivered 250 ms after the last successful S3 stimulus, and the protocol of increasing or decreasing stimulus interval repeated again.

5 IDENTIFYING REENTRY INITIATION SITES

We first analysed the activation times for each simulation protocol to determine whether an activation was caused by reentry rather than pacing. Reentry was defined as activation happening after the final pacing-induced activity for each specific vertex. We then used a graph-based approach to follow the reentrant wave back to its sources. This was done by first creating a connected graph of all mesh elements using NetworkX in Python. Each vertex in the graph was assigned its reentry activation time using the `networkx.set_node_attributes()` function. The graph was then reduced to a smaller subgraph by excluding the latest 10 ms of activations. The new subgraph was analysed to check whether it had a single or multiple connected components using the `networkx.connected_components()` function.

Multiple components indicated that reentry was initiated from multiple, separate locations. By iterating over each time-step with a 10 ms resolution, these steps were repeated in order to trace each graph component back to a cluster of 1000 connected vertices which were activated before its neighbouring clusters. The raw code for this method is available at `https://github.com/lenamyk/reentry_simulations_post_processing/blob/main/trace_graph_back_to_sources.py`.

6 SUPPLEMENTARY RESULTS

6.1 Cleft models

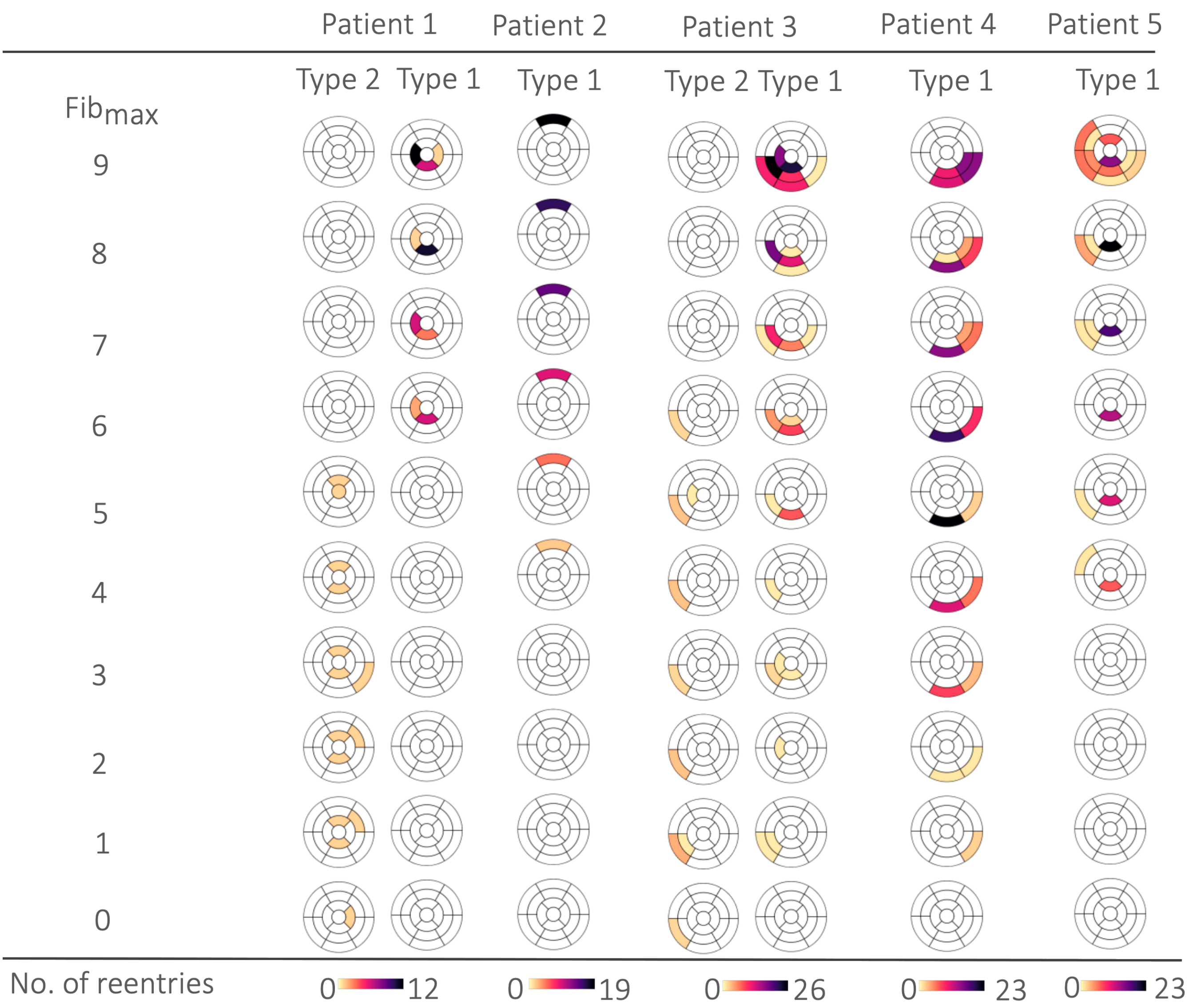

Figure S3: Cleft models with reduced conductivity and membrane changes in fibrotic regions: Number of reentries per left ventricular AHA segment for each patient and each value of fib_{max}, divided by Type 1 or 2 morphology. Higher values of fib_{max} have more Type 1-reentry and lower values more Type 2.

Multivariate regression: Type 1-reentries in clefts$_{gm}$ models

Segment-based metrics	Estimate (SE)	p-value
**Model 1**		
Max LGE intensity (%)	0.10 (0.01)	< 0.001
LGE volume (ml)	0.24 (0.11)	0.026
**Model 2**		
Mean LGE intensity (%)	0.14 (0.02)	< 0.001
LGE volume (ml)	0.47 (0.16)	0.003

Table S2. Negative binomial regression estimates for segment-specific LGE features versus number of reentries initiated in the segment. We adjusted for AHA segment, parameter choice (fib_{max} value) and patient number by including them as random effects. Only segments with LGE are included in the analysis. Model 1 AIC: 694. Model 2 AIC: 828. AIC: Akaike information criterion

6.2 Non-cleft models

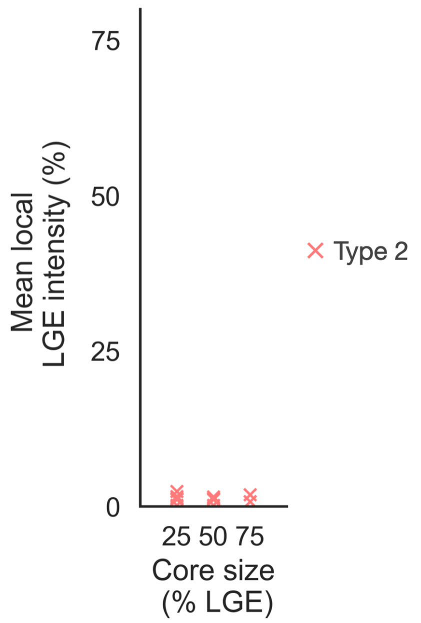

Figure S4: Mean LGE intensity in a 5 mm radius surrounding each initiation site for non-cleft models with non-conducting core tissue (non-cleft$_{nc}$). All reentries are of Type 2 morphology.

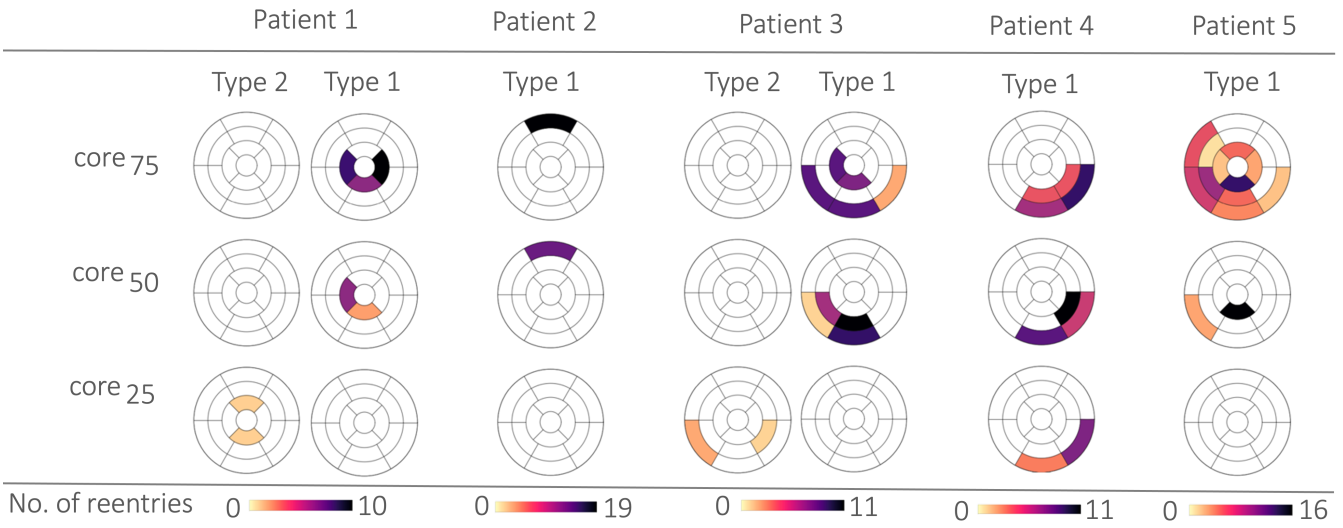

Figure S5: Non-cleft models with slowly conducting core regions: Number of reentries for non-cleft models, per left ventricular AHA segment for each patient and each value of fib_{max}, divided by Type 1 or 2 morphology. Higher values of fib_{max} have more Type 1-reentry and lower values more Type 2.

Multivariate regression: Type 1-reentries in non-cleft$_c$ models

Segment-based metrics	Estimate (SE)	p-value
Core volume (ml)	1.93 (0.81)	0.018
LGE volume (ml)	0.85 (0.27)	0.002

Table S3. Negative binomial regression estimates for segment-specific LGE features versus number of reentries initiated in the segment. We adjusted for AHA segment, parameter choice (core$_{25/50/75}$) and patient number by including them as random effects.
